# Design Optimization of Cesium Contents for Mixed Cation MA_1−x_Cs_x_PbI_3_-Based Efficient Perovskite Solar Cell

**DOI:** 10.3390/nano15141085

**Published:** 2025-07-13

**Authors:** Syed Abdul Moiz, Ahmed N. M. Alahmadi, Mohammed Saleh Alshaikh

**Affiliations:** Device Simulation Laboratory, Department of Electrical Engineering, College of Engineering and Architecture, Umm Al-Qura University, Makkah 21955, Saudi Arabia; anmahmadi@uqu.edu.sa (A.N.M.A.); msshaikh@uqu.edu.sa (M.S.A.)

**Keywords:** solar cell, perovskite solar cell, mixed-cation, MA_1−x_Cs_x_PbI_3_, caesium, power conversion efficiency, TiO_2_, simulation, Spiro-OMeTAD

## Abstract

Perovskite solar cells (PSCs) have already been reported as a promising alternative to traditional energy sources due to their excellent power conversion efficiency, affordability, and versatility, which is particularly relevant considering the growing worldwide demand for energy and increasing scarcity of natural resources. However, operational concerns under environmental stresses hinder its economic feasibility. Through the addition of cesium (Cs), this study investigates how to optimize perovskite solar cells (PSCs) based on methylammonium lead-iodide (MAPbI_3_) by creating mixed-cation compositions of MA_1−x_Cs_x_PbI_3_ (x = 0, 0.25, 0.5, 0.75, 1) for devices A to E, respectively. The impact of cesium content on the following factors, such as open-circuit voltage (V_oc_), short-circuit current density (J_sc_), fill factor (FF), and power conversion efficiency (PCE), was investigated using simulation software, with ITO/TiO_2_/MA_1−x_Cs_x_PbI_3_/Spiro-OMeTAD/Au as a device architecture. Due to diminished defect density, the device with x = 0.5 (MA_0.5_Cs_0.5_PbI_3_) attains a maximum power conversion efficiency of 18.53%, with a V_oc_ of 0.9238 V, J_sc_ of 24.22 mA/cm^2^, and a fill factor of 82.81%. The optimal doping density of TiO_2_ is approximately 10^20^ cm^−3^, while the optimal thicknesses of the electron transport layer (TiO_2_, 10–30 nm), the hole-transport layer (Spiro-OMeTAD, about 10–20 nm), and the perovskite absorber (750 nm) were identified to maximize efficiency. The inclusion of a small amount of Cs may improve photovoltaic responses; however, at elevated concentrations (x > 0.5), power conversion efficiency (PCE) diminished due to the presence of trap states. The results show that mixed-cation perovskite solar cells can be a great commercially viable option because they strike a good balance between efficiency and performance.

## 1. Introduction

In recent years, the availability of natural energy resources has dropped dramatically because of the growing energy requirements [1,2]. Many schools of thought are increasingly concerned about the future demand and supply of energy from traditional resources. Consequently, some developing countries have already switched to utilizing renewable energy sources, specifically solar energy. Silicon solar cells provide a highly efficient, cost-effective, and green option for photovoltaic energy, making them the best choice in the solar cell industry [3,4]. However, in several countries, the cost of Si-based solar cells is still higher than that of traditional energy sources per unit of energy. Hence, significant efforts are being made to produce affordable solar cells that can compete with the per-unit costs of traditional energy resources [4,5].

Perovskite solar cells have attracted a lot of interest recently because of their remarkable photovoltaic potential. These materials can be made using simple methods at temperatures below 150 °C, and they have the potential to achieve very high efficiency (over 25% PCE) while being relatively cheap to produce. Their distinct characteristics include native defect tolerance, extended charge carrier diffusion lengths (>1 μm), and exceptional light absorption coefficients (10^4^–10^5^ cm^−1^), all of which are made possible by adjustable bandgap engineering (1.2–2.3 eV) by simple compositional changes. With applications ranging from flexible, lightweight modules to high efficiency tandem combinations and semi-transparent building-integrated photovoltaics, the technology provides unparalleled adaptability. Despite the significant enhancement in solar efficiency of lead-based perovskites, which surged from 3% to 25% within a decade, continuous efforts to optimize large-scale manufacturing and elevate performance continue to address recent hurdles for commercial viability [6,7,8,9].

The insufficient durability of perovskite solar cells under operating conditions is a significant concern. When exposed to environmental factors such as humidity, high temperatures, oxygen, and sunlight, perovskite materials may degrade. These features impair solar cells’ long-term performance and give rise to serious questions about their endurance. Researchers have conducted a variety of studies to enhance the resilience of perovskite solar cells [10,11]. Encapsulating the perovskite layer to control moisture and oxygen exposure [12], carefully engineering the interfaces [13], adding particular elements or ions as dopants to the perovskite layer to improve its properties [14], carefully choosing the compositional materials in the perovskite structure that have a significant impact on its robustness [15,16], modifying the device architecture to maximize performance [17], and using natural drying techniques are just a few of the research proposals. The most economical drying technique for creating durable perovskite films for solar cells may be the deposition of perovskite thin films without the use of spin coating, antisolvent, gas, or vacuum techniques. Furthermore, using chemical or annealing treatments may improve crystallinity and decrease flaws, increasing dependability [18,19].

Significant problems continue to exist in the way of perovskite’s commercial competitiveness versus silicon photovoltaics, despite its encouraging PCE. The key issue is that perovskite solar cell efficiency is still lower than silicon’s. Even though the most effective perovskite uses minimal material, there are still environmental and regulatory obstacles to overcome. Third, compared to silicon, high-quality perovskite films free of pinholes, impurities, or breakdown routes are still difficult to scale and replicate. Economic barriers are ultimately imposed by the supply chain and costs related to materials such as Spiro-OMeTAD or gold electrodes. The perovskite industry could remain stuck in a loop of academic promise without reaching commercial viability if PCE, toxicity reduction, and scalable low-cost production do not improve.

Recently, methylammonium lead iodide (MAPbI_3_) has received the most significant attention among various research groups as an excellent perovskite photon-absorbing material utilized in high efficiency solar cells. The hybrid organic-inorganic structure of MAPbI_3_ perovskite crystal lattices is composed of organic methylammonium cation (CH_3_NH_3+_) and inorganic lead iodide (PbI_3−_) as anion [20,21]. Despite all these advantages, the major issue with MAPbI_3_ is the reactivity with water molecules and polar solvents, and it is the most obvious threat to their environmental performance. The uncertainty problem arises because of the exceedingly unstable nature of the MA site for MAPbI_3_, making it less resilient when subjected to humidity, high temperatures, and even ambient conditions. Both atomic layer deposition and suitable sealing techniques have shown a significant reduction in the moisture sensitivity of MAPbI_3_. The principal concern with MAPbI_3_ is its vulnerability to defect generation at high temperatures. Optimizing the composition of MAPbI_3_ perovskite may be one of the most promising techniques to address the challenges of electronic structure and performance [22,23,24].

Mixed cation compositions refer to the blending of different cations such as methylammonium (MA), formamidine (FA), or cesium (Cs), etc., in a specific and predetermined proportion or ratio for MAPbI_3_. This has the potential to greatly modify the properties and behaviors of MAPbI_3_. From a power conversion efficiency perspective, both MAPbI_3_ and CsPbI_3_ are excellent perovskite absorber layers. However, both these absorber layers suffer some environmental instabilities, such as humidity and thermal instability. In other words, they seriously degrade their photovoltaic response under humid and thermal environments. It is experimentally observed that incorporation of small Cs contents with organic MA for PbI_3_ absorber improves the photovoltaic responses of the perovskite layer by defect passivation. Therefore, MA_1−x_Cs_x_PbI_3_ is preferred for this study as a perovskite absorber layer due to its balanced behavior between power conversion efficiency and environmental stability [25,26]. By manipulating the types and amounts of mixed cations, it is possible to achieve desired characteristics such as enhanced stability and conductivity and/or optical properties of MAPbI_3_ [27]. The mixed composition of MA cations with Cs cations directly affects the crystal structure and properties of the MAPbI_3_ perovskite material.

For lead halide-based perovskite absorber layers, both methylammonium (MA, e.g., MAPbI_3_) and formamidinium (FA, e.g., FAPbI_3_) are the most reported organic cations. The FAPbI_3_ offers a relatively improved photovoltaic response, while MAPbI3 is a much better option for solution processability and hence high-quality thin-film deposition compared to the FAPbI_3_ perovskite layer. High-quality thin-film deposition causes minimizing defects as well as enhancing crystal uniformity, which is one of the most important requirements for highly efficient next-generation solar cells [28].

In line with the established advantages of MAPbI_3_ for carrier transport, our research demonstrates that the addition of Cs^+^ to MA_1−x_Cs_x_PbI_3_ further improves crystallinity and lowers defect density, which results in better photovoltaic performance [29]. It is well reported in literature that the larger size of Cs cations compared to MA cations can lead to a denser and more compact crystal lattice, hence reducing the potential of structural defects and improving the material’s tolerance for fluctuations in moisture and temperature [30,31], but at the same time, if the contents of Cs are not optimized, it may degrade the power conversion efficiency compared to MAPbI_3_ [32].

The photovoltaic characteristics as well as the efficiency of PbI_3_-based perovskites are greatly impacted by the existence of mixed cations (MA and Cs). MA_1−x_Cs_x_PbI_3_ materials are still an emerging class of perovskites, with little knowledge of the ideal cation compositions for high solar efficiency, despite the lack of published investigations. The doping density, thickness of each layer, and the perovskite absorber, as well as the ratio of MA to Cs, have a significant impact on important performance metrics, including fill factor, power conversion efficiency, open-circuit voltage, and short-circuit current. This study uses thorough device modeling and simulation to solve these important issues.

To achieve this objective, we suggested five variations of perovskite MA_1−x_Cs_x_PbI_3_, where x takes on the values of 0, 0.25, 0.5, 0.75, and 1, respectively. We also chose the most effective combination of electron transport and hole-transport layers, which have been previously published in literature as ITO and Spiro-OMeTAD, respectively, for (i) Device A (ITO/TiO_2_/MAPbI_3_/spiro-OMeTAD). (ii) Device B (ITO/TiO_2_/MA_0.75_Cs_0.25_PbI_3_/spiro-OMeTAD). (iii) Device C (ITO/TiO_2_/MA_0.5_Cs_0.5_PbI_3_/spiro-OMeTAD), (iv) Device D (ITO/TiO_2_/MA_0.25_Cs_0.75_PbI_3_/spiro-OMeTAD), and (v) Device E (ITO/TiO_2_/CsPbI_3_/spiro-OMeTAD), respectively.

The present investigation reports a notable advancement in mixed-cation perovskite solar cells (PSCs) via systematic optimization of cesium (Cs) composition in MA_1−x_Cs_x_PbI_3_. An optimal ratio of x = 0.50 (MA_0.5_Cs_0.5_PbI_3_) is identified, achieving a simulated power conversion efficiency (PCE) of 18.53%, which exceeds many reported experimental MA-Cs systems. The study presents several key findings: (i) a thorough investigation of five Cs concentrations, indicating that 50% Cs effectively reduces defect density and trap states while improving environmental stability; (ii) simultaneous enhancement of device architecture, featuring ultra-thin charge transport layers (TiO_2_: 10–30 nm; Spiro-OMeTAD: 10–20 nm) and a 750-nm absorber, optimizing light absorption and charge extraction; (iii) a mechanistic understanding that correlates peak performance at x = 0.50 with decreased activation energy for recombination and an improved fill factor (82.81%); and (iv) the establishment of design principles for MA-dominated compositions, differentiating from previous FA-Cs research. This study examines commercialization barriers by illustrating how controlled incorporation of Cs stabilizes the bulk and interface trap density and reduces efficiency losses at elevated Cs levels (x > 0.50), providing a framework for the development of efficient perovskite solar cells.

## 2. Device Models for Simulation

### 2.1. Simulation Software

The SCAPS-1D software, version 3.3.10, was applied for simulating the photovoltaic responses derived from the systems of equations discussed below. The SCAPS-1D, also known as the Solar Cell Capacitance Simulator-1 Dimension, is an application software used for simulating one-dimensional solar cells. The software originated at the University of Gent, Belgium’s Department of Electronics and Information Systems (ELIS). The software utilizes diverse physical models to model the functioning of solar cells, encompassing the impacts of carrier transport, recombination, and optical absorption. The software can process many material systems, including CIGS, crystalline silicon, organic/polymer thin films, perovskite thin-film solar cells, and upcoming photovoltaic devices [33,34,35,36,37,38,39,40,41].

### 2.2. Device Architecture of the Proposed Devices

**Figure 1:**
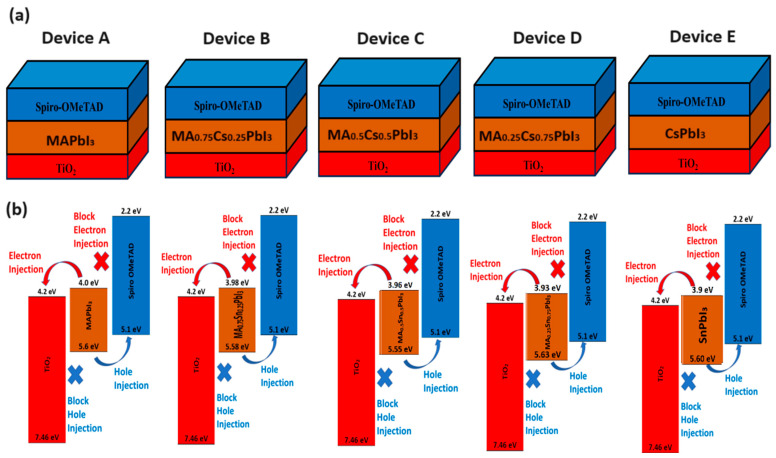
(**a**) Shows the design architecture, while (**b**) demonstrates energy band diagrams of the suggested perovskite solar cell devices, such as (i) Device A (TiO_2_/MAPbI_3_/spiro-OMeTAD). (ii) Device B (TiO_2_/MA_0.75_Cs_0.25_PbI_3_/spiro-OMeTAD). (iii) Device C (MA_0.5_Cs_0.5_PbI_3_/spiro-OMeTAD), (iv) Device D (TiO_2_/MA_0.25_Cs_0.75_PbI_3_/spiro-OMeTAD), and (v) Device B (TiO_2_/CsPbI_3_/spiro-OMeTAD), respectively.

Figure 1a shows the five different device architectures of perovskite solar cells used in this study. These devices are:

(i) Device A (TiO_2_/MAPbI_3_/spiro-OMeTAD),
(ii) Device B (TiO_2_/MA_0.75_Cs_0.25_PbI_3_/spiro-OMeTAD),
(iii) Device C (TiO_2_/MA_0.5_Cs_0.5_PbI_3_/spiro-OMeTAD),
(iv) Device D (TiO_2_/MA_0.25_Cs_0.75_PbI_3_/ spiro-OMeTAD),
(v) Device E (TiO_2_/CsPbI_3_/spiro-OMeTAD),

respectively. Every device is identical to other devices, with only the distinction being the composition of the perovskite absorber layer. Each component exhibits a traditional n-i-p configuration, in which sunlight is directed towards the ITO surface. The AM1.5G solar spectrum is commonly used for modeling purposes.

Figure 1b shows an energy level diagram for each device in the simulation. Energy levels have a considerable impact on photocarrier transmission, making them essential for the device’s functionality. Figures show that the perovskite absorber layer has a lower conduction band than TiO_2_, indicating effective electron transport. Similarly, the perovskite layer’s valence maximum is lower than that of the Spiro-OMeTAD, enabling effective hole-transport [42].

### 2.3. Simulation Materials Parameters

The reliability of the photovoltaic behavior derived from a universal device model relies on the precision of the physical, optical, electrical, and material parameters of each layer, including ITO, TiO_2_, Spiri-OMeTAD, and the perovskite absorber layer, respectively. The MA_1−x_Cs_x_PbI_3_ absorbers are relatively new types of perovskite materials, and there is not enough information available in the literature. Table 1 lists the material parameters of the ITO, TiO_2_, Spiri-OMeTAD, and perovskite absorber layers, as well as the likely range of reported thickness. We retrieved and compiled these values from relevant literature sources. The trap density characteristics are used to simulate the presence of traps or deep-level defects in the perovskite absorber material and interfaces [43]. The presence of these traps can have a considerable impact on the performance of the designed solar cells. They can accumulate and then release charge carriers, which can result in phenomena, including carrier recombination, trapping, and de-trapping.

It is well accepted that interface quality significantly influences the efficiency of perovskite solar cells, especially for charge transport and recombination losses. In this simulation, the interfaces—such as ETL/perovskite and perovskite/HTL—were modeled by integrating interface defect parameters that represent the density of trap states, their energy distribution, and carrier capture cross-sections. Table 2 shows the inclusion of neutral defect states with specific energy levels related to the valence band at both interfaces, enabling SCAPS-1D to include interface-assisted recombination losses. These traps simulate the physical consequences of grain boundary defects, suboptimal band alignment, or interfacial chemical discrepancies, all of which may impede charge extraction and diminish open-circuit voltage and fill factor. Although SCAPS is unable to comprehensively represent intricate interfacial processes such as dipole generation, interlayer diffusion, or chemical passivation, our methodology provides a first-order approximation of interfacial losses grounded in defect physics. The findings indicate that controlling defect density and doping profiles at these interfaces is essential for enhancing overall device performance, underscoring the need for meticulous material engineering and experimental validation [44,45,46,47,48,49,50,51,52,53,54,55]. 

### 2.4. Simulation Device Models

Most of the software, which is used for the simulation and modeling of the solar cells typically solves a set of semiconductor device-based integro-differential equations using traditional mathematical techniques. The overall performance matrix of solar cells, encompassing short-circuit current, open-circuit voltage, fill factor, and power conversion efficiency, is typically ascertained by solving these equations. These equations can be classified into the following groups [56,57,58,59,60,61]:

#### 2.4.1. Poisson’s Equation

The electric field and potential (ϕ) produced by excess charge (hole (p(x)), electron (n(x)), donor (N_D_), acceptor (N_A_), trapped hole (ρ_p_,), and trapped electron (ρ_n_) densities) as well as their relationship to the charge distribution inside the various solar cell layers depending on the thickness (x) is described by the Poisson’s model. Mathematically, Poisson’s equation can be defined as [56,57,58,59,60,61]

(1) $$\frac{d^{2}\phi \left(x\right)}{dx^{2}}=\frac{e}{\epsilon_{0}\epsilon_{r}}\left(p\left(x\right)-n\left(x\right)+N_{D}-N_{A}+\rho_{p}-\rho_{n}\right)$$

In this case, the absolute and relative dielectric constants of the semiconducting material in each layer are “$\epsilon_{0}$” and “$\epsilon_{r}$”, respectively, and “e” represents the electric charge (with typical value 1.602 × 10^−19^ C).

#### 2.4.2. Continuity Equation

An equation that explains the life cycle (generation (G), recombination (R)) of electrons and holes in terms of electron current density (J_n_) and hole current density (J_p_) separately is known as a continuity equation or, in some literature, known as a transport equation [56,57,58,59,60,61] and can be defined as.

(2) $$\frac{dJ_{n}}{dx}=G-R$$

(3) $$\frac{dJ_{p}}{dx}=G-R$$

#### 2.4.3. Charge Transport Model

The proposed devices ITO/TiO_2_/MA_1−x_Cs_x_PbI_3_/Spiro-OMeTAD/Au are simply p-i-n diodes. The semiconductor p-i-n junction diode is the basic building block of any solar cell. Therefore, to simulate the photovoltaic response, any charge transport models that are appropriate to p-i-n junction diodes may be utilized, and one of the most common models of them is listed below. The standard charge transport model of the p-i-n junction diode posits that the overall current is the sum of the current densities of electrons and holes and that each current density reflects the cumulative effects of both drift and diffusion currents [56,57,58,59,60,61].

(4) $$J=J_{n}+J_{p}$$

(5) $$J_{n}=D_{n}\frac{dn}{dx}+\mu_{n}n\frac{d\phi }{dx}$$

(6) $$J_{p}={-D}_{p}\frac{dp}{dx}+\mu_{p}p\frac{d\phi }{dx}$$

Here µ_n_ and µ_p_ are the mobilities of electron and hole, characterize the drift current. While D_n_ and D_p_ are the diffusion coefficients of electron and hole density, characterize the diffusion current. 

#### 2.4.4. Photon Absorption Model

Out of all the photoabsorption models that SCAPS-1D deals with the conventional optical absorption model is chosen for this study and is shown in Equation (7). According to this conventional model, the optical absorption coefficient “α” is defined as “α(λ)” and is dependent on the optical wavelength “λ” with energy “hν”. Equation (7) states that in this model, both constants (A and B) are arbitrary in nature, and “$E_{g}$” stands for the energy bandgap of the relevant thin-film layer, and all these variables are interrelated as [61].

(7) $$\alpha (\lambda )=\left(A+\frac{B}{h\nu }\right)\sqrt{h\nu -E_{g}}$$

### 2.5. Simulation Steps

The SCAPS-1D simulation process consists of a series of actionable tasks that must be performed sequentially. It is important to note that both ETL and HTL are optimized with respect to thickness and doping density prior to the optimization of the perovskite absorption layer. For optimal power conversion efficiency, the following simulation algorithm is recommended for this study.

- Step 1: Set up the simulation environment on SCAPS-1D. First, set up the simulation by defining the layers of the device along with their respective standard environments, geometries, and physical parameters to initialize the software.
- Step 2: Extract the parameter information of novel parameters from literature, by examining the literature [44,45,46,47,48,49,50,51,52,53,54,55], determine the optimal physical and material input parameters for the perovskite absorbing layer as well as the charge transport layers necessary for executing a significant simulation.
- Step 3: Roughly assess the ranges of various parameters to initialize the simulation process and recommend the thickness range and doping density for each layer of the devices for each of the considered devices as per literature.
- Step 4: Roughly estimate the typical values for various physical and material parameters to initialize the simulation process, recommend the estimation of initial values for various physical and material parameters to initialize the simulation process for each layer of the device for each of the considered devices. This is the most time-consuming process.
- Step 5: Optimize and assess photovoltaic parameters as a function of electron transport layer’s thickness; execute multiple rounds of simulations to ascertain the optimal photovoltaic characteristics for all devices as a function of TiO_2_ thickness.
- Step 6: Optimize and assess photovoltaic parameters as a function of electron transport layer’s doping density; determine each device’s optimal photovoltaic properties as a function of TiO_2_ doping density by running several simulations.
- Step 7: Optimize and assess photovoltaic parameters as a function of hole-transport layer thickness; conduct a series of simulations to ascertain the optimal photovoltaic characteristics based on the thickness of spiro-OMeTAD.
- Step 8: Optimize and assess photovoltaic parameters as a function of hole-transport layer doping density; conduct a series of simulations to ascertain the optimal photovoltaic characteristics of each device, based on the doping density of spiro-OMeTAD.
- Step 9: Optimize and assess photovoltaic parameters as a function of absorber layer thickness for each device. Conduct a series of simulations to ascertain the optimal thickness of the perovskite absorber layer for each device. This process aims to identify the configuration that yields the highest power conversion efficiency and quantum efficiency. Subsequently, revise to incorporate the optimal thickness of the absorber for subsequent simulations.
- Step 10: Determine the final photocurrent-voltage response and parameters of the optimal devices by performing a series of simulations to determine the photovoltaic current-voltage response and other photovoltaic parameters of all the optimal devices of each device.
- Step 11: Estimate the final photovoltaic parameters of the optimal photovoltaic devices and calculate the ultimate photovoltaic parameters, especially power conversion efficiency for all optimal devices.
- Step 12: The simulation is terminated.

## 3. Results and Discussion

### 3.1. Thickness Optimization of Electron Transport Layer

The thickness of the ETL in any perovskite solar cell plays a very important role in multiple aspects, such as (i) charge extraction at the cathode, (ii) charge transport, (iii) optical response, (iv) charge recombination, (v) interfacial properties, and (vi) mechanical flexibility. Generally, decreasing the thickness of the electron transport layer improves the ability of light to pass through the perovskite layer, leading to enhanced optical absorption and hence the production of photocurrent. Conversely, increasing the thickness of the electron transport layer improves the efficiency of charge transport, but it may also result in increased optical losses [62,63,64].

**Figure 2:**
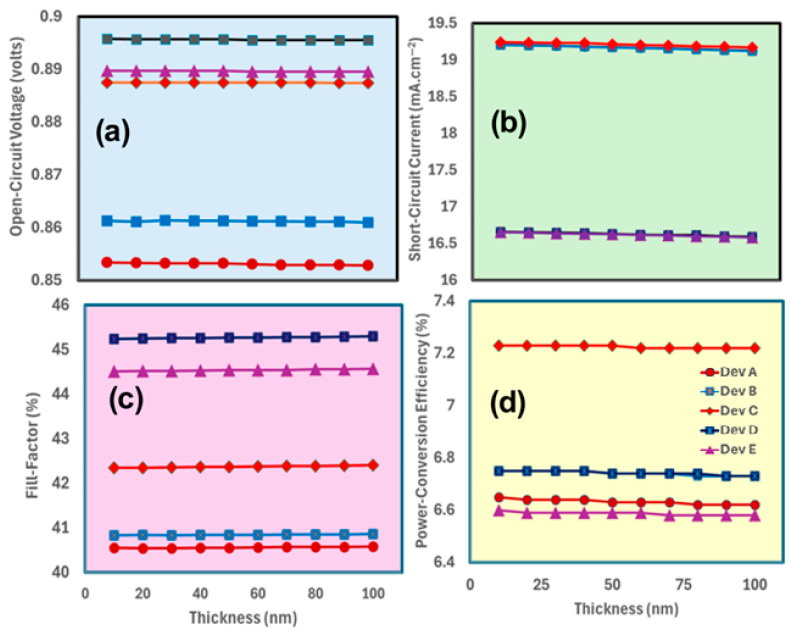
Displays the photovoltaic parameters such as (**a**) open-circuit voltage, (**b**) short-circuit current, (**c**) fill factor, and (**d**) power conversion efficiency of MA_1−x_Cs_x_PbI_3_-based devices as Device A, Device B, Device C, Device D, and Device E, respectively, by the increasing function of the electron transport layer thickness.

Optimizing the electron transport layer thickness is crucial for achieving high photovoltaic responses in MA_1−x_Cs_x_PbI_3_-based perovskite solar cells [65], as discussed above. The open-circuit voltage results as shown in Figure 2a reveal that for all five devices (A, B, C, D, and E), the open-circuit voltage is nearly constant over a large range of TiO_2_ thicknesses. For Device “A” ITO/TiO_2_/MAPbI_3_/spiro-OMeTAD (x = 0), the open-circuit voltage has approximately remained constant at ~0.85 V for TiO_2_ thicknesses ranging from 10 to 100 nm, with only a very small voltage drop observed at the thickest point (100 nm). Over the whole thick range, the open-circuit voltage values for all other devices, such as B (x = 0.25), C (x = 0.5), D (x = 0.75), and E (x = 1), stay within a narrow range of 0.86–0.89 V, following a very similar trend. In a very similar fashion, the results for Figure 2b show that the short-circuit current values of devices A, B, C, D, and E demonstrate a slight decrease as compared to the thickness of TiO_2_ increasing. The current density for Device A (x = 0) starts from 19.2 mA/cm^2^ at a thickness of 10 nm and decreases to 19.1 mA/cm^2^ at a thickness of 100 nm. Based on these results, it can be inferred that a TiO_2_ layer about 10 nm thick is best for obtaining the optimal short-circuit current in MA_1−x_Cs_x_PbI_3_-based perovskite solar cells.

For the fill factor (see Figure 2c), the values remain relatively stable across the thickness range for all five devices (A, B, C, D, and E). Device A (x = 0) has a fill factor around 40.56, Device B (x = 0.25) around 40.83, Device C (x = 0.5) around 42.35, Device D (x = 0.75) around 45.24, and Device E (x = 1) around 44.51. This suggests that the FF is not highly sensitive to the TiO_2_ thickness in these MA_1−x_Cs_x_PbI_3_ devices. Finally, the power conversion efficiency results in Figure 2d show that among all devices, Device C (x = 0.5 for MA_1−x_Cs_x_PbI_3_) represents the highest efficiency as a function of electron transport layer, reaching a maximum of 7.23% at 10 nm thicknesses. Therefore, determining the optimal thickness of TiO_2_ requires careful optimization for each specific perovskite solar cell layer in the system, as it involves finding a balance between these conflicting factors, as discussed above. The results clearly demonstrate that the performance of devices A-E was influenced by the thickness of the TiO_2_ ETL within the optimal range of 10–30 nm.

Although experimental investigations often indicate TiO_2_ thickness of approximately 100 nm, our simulation-based research proposes a narrower range of 10–30 nm. Such discrepancy often arises between the theoretical simulation outcomes and empirical measurements for TiO2 as well as MA_1−x_Cs_x_PbI_3_, attributable to its variables such as non-homogeneous cation distribution, flaws, and interface irregularities in actual devices. Because simulations use mathematical models based on ideal circumstances, which naturally presume uniform thickness, doping density, and material properties—elements that often exhibit considerable variation in actual thin-film deposition processes. These idealized assumptions enable models to forecast the maximum potential of photovoltaic performance; nevertheless, they may not adequately reflect the real discrepancies seen during manufacturing. Nonetheless, device modeling and simulation have been essential to semiconductor research from its origin, surmounting initial obstacles of inadequate software, hardware, and knowledge. Currently, this topic has evolved into a recognized discipline, bolstered by sophisticated computational tools that foster innovation in electronics and photovoltaics [66], despite ongoing discrepancies between simulated and experimental outcomes attributable to the idealized characteristics of theoretical models.

### 3.2. Doping Optimization of Electron Transport Layer

In a similar way, the optimization process for the doping density of TiO_2_ is also essential for improving the charge extraction, charge transport, and hence interfacial properties of solar cells. Which ultimately leads to an improvement in the overall power conversion efficiency. On the other hand, the doping of TiO_2_ involves many other physical and chemical processes, which make doping of TiO_2_ not only very complex but also challenging [67,68,69].

- The defect chemistry of TiO_2_ is highly complex, involving various types of point defects and their interactions with the dopants.
- In most of the cases, the incorporation of dopant atoms into the TiO_2_ lattice structure is often limited by the maximum concentration that can be achieved without causing adverse effects, such as the formation of structural distortions. Surpassing the maximum limitations can lead to the degradation of the required characteristics and responses [68,69].

**Figure 3:**
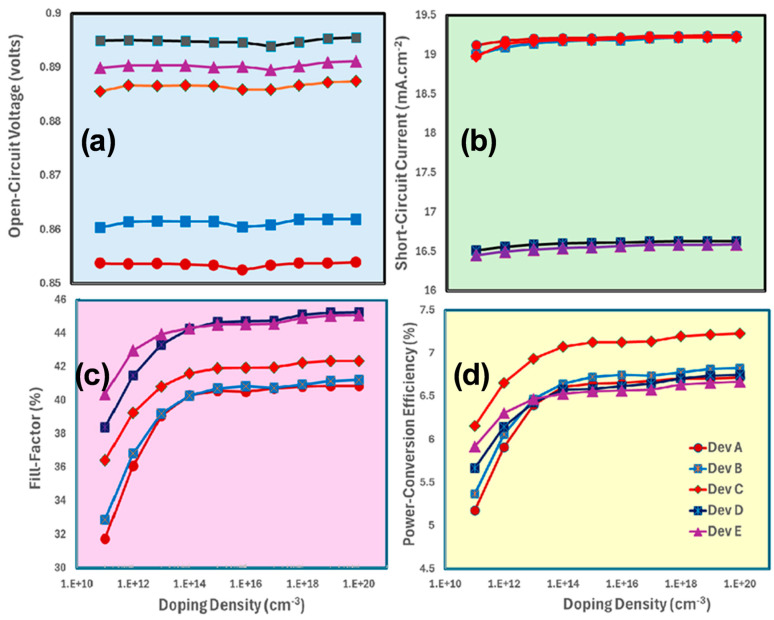
Demonstrates the photovoltaic parameters such as (**a**) open-circuit voltage, (**b**) short-circuit current, (**c**) fill factor, and (**d**) power conversion efficiency of MA_1−x_Cs_x_PbI_3_-based devices as Device A, Device B, Device C, Device D, and Device E, respectively, by the increasing function of the doping density of TiO_2_.

Figure 3a–d shows the photovoltaic responses of MA_1−x_Cs_x_PbI_3_-based all devices as a function of the doping density of TiO_2_. These responses are open-circuit voltage (a), short-circuit current (b), and fill factor (c), as well as overall power conversion efficiency (d) for given MA_1−x_Cs_x_PbI_3_-based photovoltaic devices. Regardless of the device configuration (Dev A to Dev E), Figure 3 shows that approximately these photovoltaic parameters, especially the power conversion efficiency of perovskite solar cells, reach their maximum values within the doping density range of 10^19^ to 10^20^ cm^−3^, respectively.

According to the results as shown in Figure 3a–d, the solar cell’s power conversion efficiency is maximized at the doping density of about 10^20^ cm^−3^ for the electron -transport-layer. Such high doping density lowers the electrical resistance or increases the electron transport layer’s conductivity, which in turn leads to a higher free charge carrier concentration and overall enhances charge extraction from the active layer. Additionally, a larger potential difference between layers is produced by the higher doping, which helps for the efficient separation of the photogenerated electron-hole pairs. The most effective charge extraction to the electron transport layer is made possible by this balance of charge carrier concentration, conductivity, and inherent potential. Additionally, the enhanced conductivity lowers series resistance, increasing the solar cell’s fill factor and overall efficiency [68,69].

### 3.3. Thickness Optimization of Hole-Transport Layer

**Figure 4:**
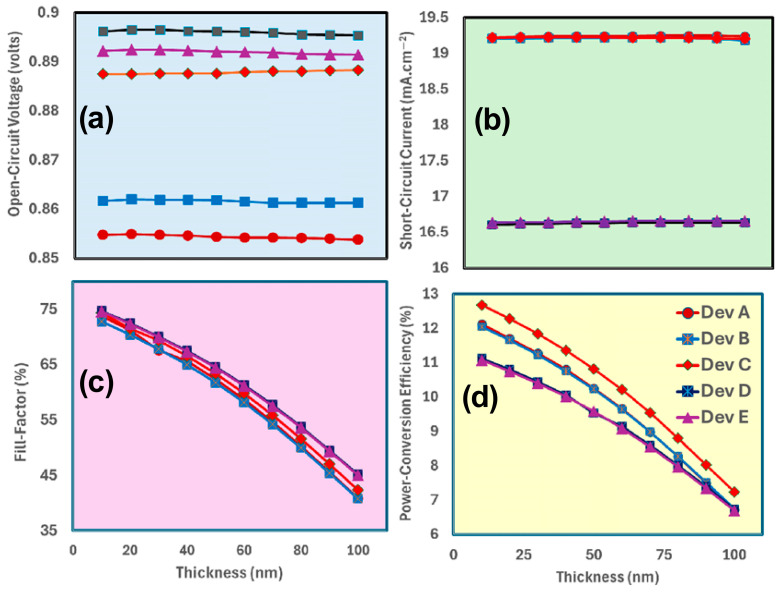
Illustrate the photovoltaic parameters, i.e., (**a**) open-circuit voltage, (**b**) short-circuit current, (**c**) fill factor, and (**d**) power conversion efficiency of MA_1−x_Cs_x_PbI_3_-based devices as Device A, Device B, Device C, Device D, and Device E, respectively, by the increasing function of spiro-OMeTAD thickness.

Figure 4a–d demonstrate the photovoltaic performance of MA_1−x_Cs_x_PbI_3_-based perovskite solar cells, for Device A (pure MAPbI_3_), Device B (MA_0.75_Cs_0.25_PbI_3_), Device C (MA_0.5_Cs_0.5_PbI_3_), Device D (MA_0.25_Cs_0.75_PbI_3_), and Device E (pure CsPbI_3_) as a function of spiro-OMeTAD hole-transport layer (HTL) thickness (0–100 nm). Figure 4a illustrates the open-circuit voltage, which is uniform across all devices, varying from 0.85 V to 0.90 V, with Device E exhibiting the highest open-circuit voltage at 0.89 V, signifying improved voltage output with pure CsPbI_3_. Figure 4b illustrates the short-circuit current density, with Device A displaying the highest values (19–19.5 mA/cm^2^), while an increase in Cs content leads to a reduction (Device D at approximately 16.5 mA/cm^2^), indicating that Cs substitution may introduce defects or reduce light absorption, although J_sc_ remains predominantly unaffected by HTL thickness.

Figure 4c,d demonstrate that both the fill factor and power conversion efficiency significantly decrease as the thickness of spiro-OMeTAD increases [70,71,72,73]. The fill factor diminishes from 75% (Device A at 0 nm) to roughly 45% (at 100 nm), whereas Device D consistently displays the lowest FF at 35% (at 100 nm). This trend demonstrates the adverse impact of increased Cs content and thicker hole-transport layers on charge extraction due to elevated series resistance. The PCE decreases from 12.5% (Device A at 10 nm) to roughly 7.5% (at 100 nm), with Device D displaying the lowest PCE of 6% at 100 nm. This indicates that pure MAPbI_3_ with a minimal HTL attains optimal performance, while greater HTL thickness and heightened Cs substitution diminish efficiency.

The data reveals that ITO/TiO_2_/MA_0.5_Cs_0.5_PbI_3_/spiro-OMeTAD (Device C) with a thin spiro-OMeTAD layer (~10–20 nm) achieves optimal photovoltaic performance, enhancing J_sc_, FF, and PCE. Increasing Cs content reduces overall efficiency, likely due to defects passivation, while thicker HTL layers elevate series resistance, significantly decreasing FF and PCE in all devices. To optimize perovskite solar cells, it is crucial to minimize HTL thickness and precisely control Cs incorporation to improve charge transport and hence device performance.

Simulation results indicate that an optimal Spiro-OMeTAD thickness of 10–20 nm and a TiO_2_ layer thickness of 10–30 nm enhance perovskite solar cells by significantly reducing series resistance, promoting efficient charge transfer, and minimizing recombination losses, as evidenced by SCAPS-1D and drift-diffusion modeling. Experimental devices may need somewhat thicker Spiro-OMeTAD layers (≥20 nm) and tailored TiO_2_ thickness (≥10 nm) to guarantee homogeneous coverage and avert direct contact between the perovskite and the electrode. Conversely, models may presume an ideal morphology, endorsing a thickness range of 10–30 nm for both materials, which addresses low resistance with efficient charge extraction while ignoring pinhole flaws inherent in real production. The 10–30 nm range for Spiro-OMeTAD and TiO_2_ is considered appropriate and beneficial for theoretical optimization research; yet, practical applications may need some modifications to ensure scalability.

### 3.4. Doping Optimization of Hole-Transport Layer

**Figure 5:**
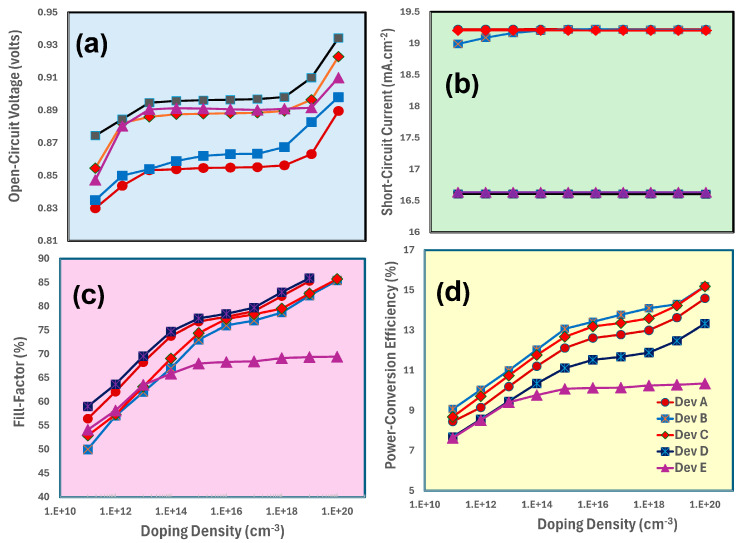
Illustrate the photovoltaic parameters, i.e., (**a**) open-circuit voltage, (**b**) short-circuit current, (**c**) fill factor, and as well as (**d**) power conversion efficiency of MA_1−x_Cs_x_PbI_3_-based all devices such as Device A, Device B, Device C, Device D, and Device E, respectively, by the increasing function of spiro-OMeTAD doping density.

Figure 5a–d demonstrates the specific responses of photovoltaic parameters: (a) open-circuit voltage, (b) short-circuit current, (c) fill factor, and (d) power conversion efficiency (PCE) for five devices fabricated from mixed halide perovskite materials, as affected by different doping densities of spiro-OMeTAD. Devices B through E integrate varying amounts of cesium into the perovskite framework, whereas Device A utilizes pure MA (MAPbI_3_) as the benchmark. Distinctly similar patterns are evident in the photovoltaic parameters of each device relative to HTL doping density. The open-circuit voltage and short-circuit behaviors stabilize with elevated Cs concentration, indicating that Cs incorporation may improve the electrical properties and structural integrity of the perovskite layer. This stabilization may improve the efficacy of solar cells by enhancing charge transport and diminishing recombination losses.

Furthermore, the power conversion efficiency and fill factor metrics offer essential insights into the overall effectiveness of these devices. In devices with high Cs content, both the fill factor and power conversion efficiency improve as the doping density of the spiro-OMeTAD hole-transport layer increases. This suggests that increased doping promotes enhanced hole mobility, which is crucial for effective charge extraction. Devices featuring a balanced MA-Cs composition, such as Device C, demonstrate optimal performance, underscoring the significance of material engineering in the progression of highly efficient perovskite solar cells. The results demonstrate that significant improvements in performance and efficiency can be achieved through careful composition and doping modifications [71,74,75,76,77,78,79].

### 3.5. Thickness Optimization of Absorber Layer (MA_1−x_Cs_x_PbI_3_)

**Figure 6:**
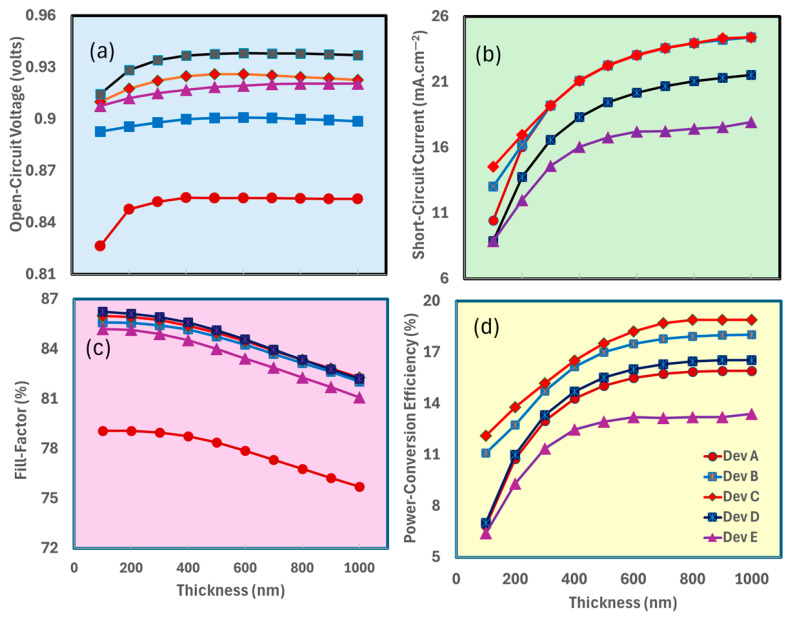
Displays the photovoltaic parameters, i.e., (**a**) open-circuit voltage, (**b**) short-circuit current, (**c**) fill factor, and as well as (**d**) power conversion efficiency of MA_1−x_Cs_x_PbI_3_-based devices such as Device A, Device B, Device C, Device D, Device E, respectively, by the increasing function of the thickness of MA_0.25_Cs_0.75_PbI_3_ (perovskite absorber layer).

Figure 6 demonstrates the simulation of several photovoltaic parameters in relation to the thickness of MA_1−x_Cs_x_PbI_3_-based perovskite films. In Figure 6a, the open-circuit voltage exhibits a progressive decrease with increasing thickness, indicating that thinner films may be advantageous for achieving larger voltages. Figure 6b,d depict the short-circuit current and power conversion efficiency, respectively, with both of these parameters attaining their maximum at a thickness of 750 nm before thereafter declining at an insignificant pace for all devices, particularly devices A to D. Among all devices, the device C with MA_0.5_Cs_0.5_PbI_3_ composition attains its peak power conversion efficiency at 750 nm, signifying an ideal equilibrium between charge carrier production and recombination at this thickness. The optimal PCE at 750 nm for MA_0.5_Cs_0.5_PbI_3_ indicates that this thickness optimizes absorption of light and charge extraction while reducing losses. Thinner films may have inadequate absorption of light, whereas thicker films may elevate recombination losses. The relationship between open-circuit voltage, short-circuit current, fill factor, and power conversion efficiency highlights the need for precise thickness to achieve high-performance devices.

Table 3 compares our predicted outcomes for the MA_0.5_Cs_0.5_PbI_3_-based perovskite solar cell with published experimental results on other mixed-cation perovskite devices. Research studies utilizing FA-Cs or MA-Cs compositions usually attain power conversion efficiencies (PCEs) between 14–17% with a V_oc_ of up to 1.25 V; however, our simulation reveals a PCE of 18.53% and a J_sc_ of 24.22 mA/cm^2^, suggesting significant potential for performance enhancement via compositional optimization and refined device architecture.

### 3.6. Photovoltaic Performance vs. Cs Content in MA_1−x_Cs_x_PbI_3_

**Figure 7:**
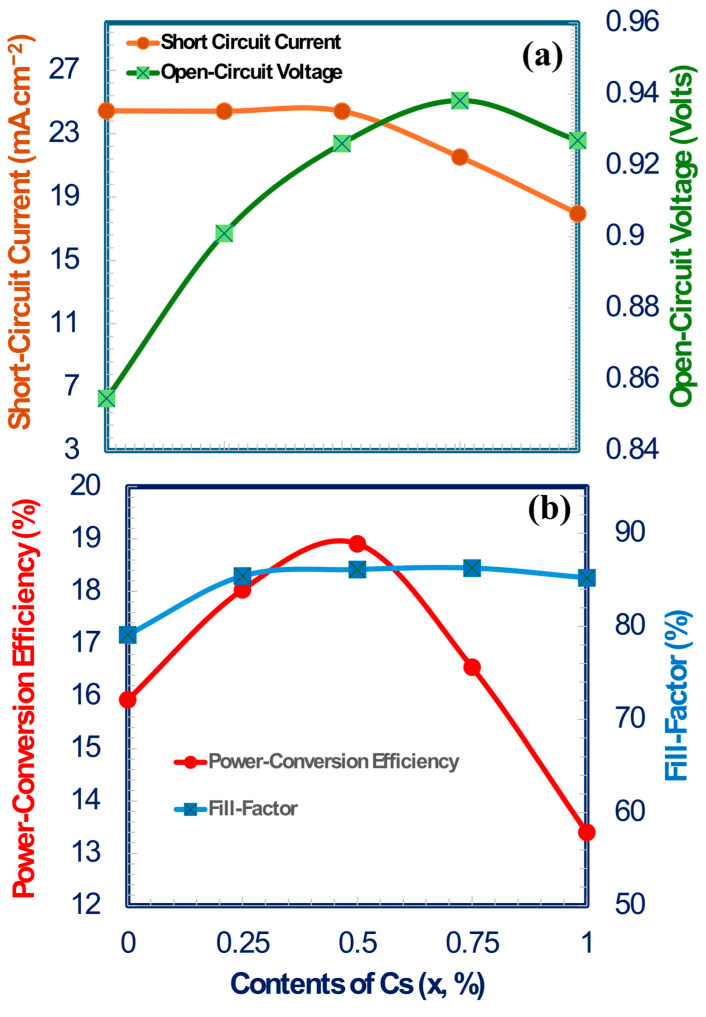
Photovoltaics performance parameters of a solar cell as a function of cesium content (x, %): (**a**) short-circuit current (mA/cm^2^) and open-circuit voltage (V); (**b**) power conversion efficiency (PCE, %) and fill factor (%) as a function of Cs content for Device C, respectively.

Figure 7a,b illustrate the variation of photovoltaic characteristics as a function of cesium concentration in the perovskite absorber layer of MA_1−x_Cs_x_PbI_3_. The photovoltaic characteristics and, therefore, power conversion efficiency of solar cells are significantly influenced by variations in cesium concentration within the perovskite composition MA_1−x_Cs_x_PbI_3_. The open-circuit voltage rises to a certain concentration of x and thereafter declines, as seen in Figure 7a, possibly owing to effects of defect density leads to the thin film quality.

In contrast, the short-circuit current density stays relatively constant at 23–24 mA/cm^2^. At x = 0.5, the highest power conversion efficiency is about 18.53%, which matches with high fill factor values close to 82.81%, showing that charge transfer and collection are working well, as seen in Figure 7b. Conversely, when the Cs content exceeds x > 0.5, particularly at x = 0.75 and x = 1, we observe a substantial drop in short-circuit current, an insignificant reduction in open-circuit voltage and fill factor, and a decrease in PCE. For Device E (x = 1), the drop in short-circuit current is substantial, reaching roughly 13%. Since pure CsPbI_3_ shows a decline in performance, it is believed to trap more states in compositions with a lot of Cs content. Therefore, to make an efficient MA_1−x_Cs_x_PbI_3_-based perovskite structure for photovoltaic applications, it’s very important to replace a moderate amount of Cs with MA structure (especially x = 0.5), but too much Cs can harm how well the device performs. Table 4 displays a clear summary of how different amounts of Cs can affect the main performance of MA_1−x_Cs_x_PbI_3_ perovskite solar cells. The devices short-circuit current, open-circuit voltage, fill- factor, and power conversion efficiency are all at their peak when x = 0.5 [89,90,91,92].

We shall now examine how a concentration of C = 50% yields the most efficient photonic response. Literature indicates that the open-circuit voltage of a solar cell is directly correlated with the device’s photovoltaic parameters as.

(8) $$V_{OC}=\frac{E_{g}}{q}-\frac{nkT}{q}\ln\left(\frac{J_{SC}}{J_{0}}\right)$$

It is a quasi-linear equation in terms of T, where A represents the y-intercept and B denotes the x-intercept.

(9) $$V_{OC}=A-BT$$

**Figure 8:**
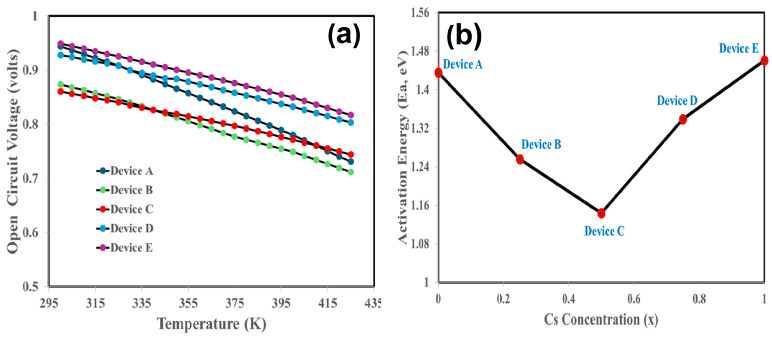
(**a**) Open-circuit voltage as a function of temperature for devices A, B, C, D, and E, respectively. (**b**) Activation of energy derived from the y-intercept of (**a**) as a function of Cs concentration (x).

Under continuous illumination (AM 1.5G, or W = 1000 W/m^2^), a quasi-linear relationship between V_oc_ and temperature is seen, as shown in Figure 8a. This clearly demonstrates two linear regions: the high temperature zone, dominated by intrinsic recombination, and the low-temperature region, closely correlated with trap-assisted recombination. Thus, the y-intercept (T ~ 0 K) of this equation helps in estimating the activation energy Ea (eV) = q V_oc_ for the specified solar cell [86,93].

(10) $$V_{OC}\left(T\;\underset{Y-Intercept}{\to }\;0\right)=E_{a}=\frac{E_{fn}-E_{fp}}{q}$$

The y-intercept for each device is estimated, which yields Ea, whereas Ea as a function of Cs concentration is illustrated in Figure 8b. It is well accepted that if Ea is almost equal to the absorber’s optical bandgap (Ea ≈ Eg), it indicates that radiative band-to-band recombination is the primary loss mechanism. Conversely, when Ea is smaller than Eg (Ea < Eg), it signifies that non-radiative trap-assisted recombination, especially via Shockley–Read–Hall (SRH) processes involving defects or mid-gap states, predominates. Figure 8b clearly demonstrates that device C has the lowest activation energy (Ea). Now it can infer that the improved power conversion efficiency at 50% content of Cs in MA_0.5_Cs_0.5_PbI_3_ may be due to the lowest non-radiative trap-assisted recombination loss.

In Figure 7a, the open-circuit voltage keeps rising, but the short-circuit current density decreases when the Cs concentration rises over 50% to 75%. The increase in open-circuit voltage may be attributed to decreased recombination losses and enhanced charge separation. In contrast, the short-circuit current is influenced by many complex factors such as recombination losses, energy bandgap, light absorption coefficient, light reflection, generation of electron-hole pairs, and other charge transport parameters (mobility, built-in potential, etc). Out of all these, the energy bandgap (Eg = 1.6 eV at x = 0.5 and 1.7 eV at x = 0.75; see Table 1) is the key factor that has the most impact on improving open-circuit voltage and decreasing short-circuit current at x = 0.5. This is primarily because of the larger band gap, which lowers losses and permits a higher open-circuit voltage. While the short-circuit current (J_sc_) decreases simultaneously because the wider band gap absorbs fewer low-energy photons, which lowers the total current produced by light. This indicates that parameters beyond trap density influence the observed rise in open-circuit voltage and corresponding decrease in short-circuit current from x = 0.5 to 0.75. When x = 1, recombination losses rise sharply, resulting in a drop in both open-circuit voltage and short-circuit current (refer to Figure 7a) [61,86].

So, it can be inferred from the results as discussed above that Device C, which contains 50% caesium, achieves a relatively improved balance between efficiency and may offer long-term operational performance as reported in various literature [93,94,95,96].

The incremental addition of Cs contents (up to 0.5) for simple MAPbI_3_ to MA_0.5_CS_0.5_PbI_3_-based perovskite layer improves the overall photovoltaic performance, as discussed above. Such improvements may be due to the reduction of the trap stats and hence optimization of the charge transport process. However, all these improvements are a direct function of Cs composition, and an excessive amount of Cs causes recombination losses, which in turn reduces the overall short-circuit current, fill factor and open-circuit voltage. To attain a balance between efficiency and performance in mixed-cation perovskite solar cells, it is essential to identify and sustain an optimal Cs/MA ratio of ~1 [97].

Generally, temperature cycling of MAPbI_3_ results in a significant decrease in open-circuit voltage due to the unstable nature of methylammonium (MA) cations, phase transitions, and ion migration, leading to increased defect formation and non-radiative recombination. In contrast, MA_0.5_Cs_0.5_PbI_3_, with 50% cesium incorporation, has enhanced defect passivations, lesser phase transition effects, and reduced ion migration, leading to a lesser reduction in open-circuit voltage. Furthermore, layer- or grain-boundary recombination can substantially influence the ideal device architecture for both materials, as heightened recombination at interfaces or grain boundaries may require improved passivation or altered layer arrangements to reduce V_oc_ losses and sustain efficiency [98].

Under AM1.5G temperature and humidity circumstances (usually 25 °C and 50–60% relative humidity, according to conventional solar measurements), the V_oc_ behavior of MAPbI_3_ and MA_0.5_Cs_0.5_PbI_3_ devices would deviate from idealized SCAPS models due to actual environmental stresses. MAPbI_3_ is extremely vulnerable to moisture-induced degradation, wherein humidity hastens the decomposition of methylammonium (MA) cations and the migration of iodide, resulting in accelerated defect formation and a more significant decrease in V_oc_ (potentially 15–25% with prolonged exposure) than SCAPS predictions, which typically presume negligible environmental interaction. In contrast, MA_0.5_Cs_0.5_PbI_3_, where cesium improves lattice crystal structure, demonstrates increased resilience to humidity and thermal stress, leading to a reduced V_oc_ drop (e.g., 5–12%) under AM1.5G conditions. Moreover, ultraviolet exposure—frequently excluded from simulations—can accelerate photo-induced deterioration in MAPbI_3_ by producing reactive species that disrupt the perovskite structure, while the introduction of Cs in MA_0.5_Cs_0.5_PbI_3_ somewhat alleviates this impact. Both materials, however, encounter heightened layer- or grain-boundary recombination in humid conditions, as moisture infiltration intensifies trap states, thereby requiring effective encapsulation or passivation (e.g., PMMA or PAN) to achieve performance levels predicted by SCAPS, with MA_0.5_Cs_0.5_PbI_3_ continuing to surpass MAPbI_3_ owing to its enhanced intrinsic defect passivation.

### 3.7. Overall Photovoltaic Response

**Figure 9:**
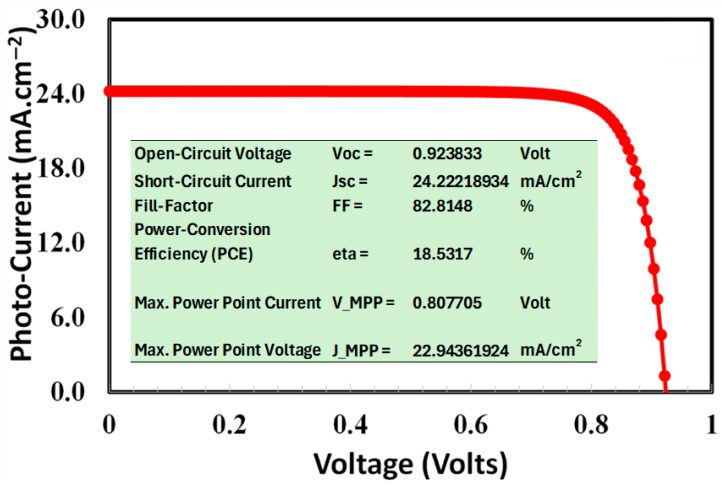
The photo current-voltage response of the most efficient Device C (ITO/TiO_2_/MA_0.5_Cs_0.5_PbI_3_/spiro-OMeTAD). The inset table of the figure shows the performance metrics derived from the J-V curve are as follows: open-circuit voltage of 0.92 V, short-circuit current density of 24.22 mA/cm^2^, fill factor of 82.81%, and power conversion efficiency of 18.53%, respectively.

Figure 9 depicts the current-voltage characteristics of Device C, a highly efficient and fully optimal perovskite solar cell comprising 50% cesium (Cs) in its composition. Several critical performance metrics are determined and discussed from these results [65,87,99,100,101,102,103]:

(i) Open-Circuit Voltage (*V*_oc_) = 0.9238 V: Figure 9 and its inset table indirectly reveal the device’s ability to separate free charge carriers without an external circuit. As a result, fewer charge carriers combine back together when the open-circuit voltage is higher, meaning there’s a lower chance of electrons and holes recombining before they reach the electrodes. to the results, it can be argued that adding cesium may stabilize the crystal lattice and lower trap states that enhance its electrical and photovoltaic properties of the perovskite layer [65,80,87].
(ii) Short-Circuit Current Density = 24.22 mA/cm^2^.

This value indicates the current produced per unit area under standard illumination when the cell is short-circuited (i.e., voltage = 0). The elevated J_sc_ value indicates effective photon absorption and charge production, which may be ascribed to [85,101,102]:

- Improved film morphology (smoother and more uniform layers)
- Minimized grain boundaries, which restrict charge recombination and trap states
- Enhanced charge transport channels within the active layer.

(iii) Fill Factor = 82.81%

The fill factor quantifies the proximity of the actual output power to the theoretical maximum, which is the product of V_oc_ and J_sc_. A high fill factor offers many advantages, such as [89,90,91,92]:

- Reduced series resistance, which indicates negligible energy loss during charge transmission.
- Effective charge extraction, which indicates superior interface quality and reduced recombination
- Enhancement is likely attributable to increased crystallinity and defect passivation resulting from Cs doping, which produces a more compact and well-aligned perovskite layer. The noted orientation-induced improvement in our results, whereby Cs^+^ doping enhances crystallographic defect passivation in MA_1−x_Cs_x_PbI_3_, attains an 82.81% fill factor via better carrier extraction [104].

(iv) Power Conversion Efficiency = 18.53%.

The PCE brings together all the other photovoltaic parameters to show how well the solar cell can turn sunlight into electricity. The high-power conversion efficiency (PCE) of Device C suggests that it offers a relatively good balance between many electrical, optical, and photovoltaic parameters, leading to efficient performance. Although an increase in Cs content (but less than x < 0.5) often enhances interface mobility and other charge transport parameters, on the other hand, excessive amounts of Cs (x > 0.5) may broaden the trap density, thereby diminishing light absorption and, hence, efficiency. At 50% cesium content, Device C attains an optimal balance, sustaining superior performance [65,102,103,104,105].

(v) Characteristics of Maximum Power Point (MPP) = 0.807 V, 22.94 mA·cm^−2^

The MPP denotes the point on the J-V curve at which the product of current and voltage reaches its maximum value. The advantageous MPP attributes of Device C suggest [78,79]:

- Effective energy extraction
- Optimal internal conductivity
- Prolonged carrier lifespan, facilitating a greater number of charges to arrive at the electrodes prior to recombination.

(vi) The Key Role of Cesium in Improving Photovoltaic Performance: The partial replacement of methylammonium (MA) with cesium tends to improve the perovskite’s energy bandgap, which in turn enhances the perovskite’s suitability for solar applications [65,102,103].

Despite the widespread recognition of Cs’s ability to improve the defect passivation of perovskite absorbers, our simulations indicate that the maximal PCE in MA_1−x_Cs_x_PbI_3_ occurs at x = 0.5. Performance deteriorates beyond this point because of Cs material-related formation of trap density. At room temperature, the transition to the non-photoactive δ-phase is a well-documented phenomenon that compromises crystallinity and optical absorption. This phenomenon is often induced by an excessive amount of Cs content Furthermore, the increase in Cs levels leads to the reduction of carrier mobility and PCE by promoting interfacial recombination and deep-level traps.

We acknowledge that the simulation does not capture multi-dimensional effects such as ion migration or phase segregation, even though these outcomes were modeled in SCAPS-1D using composition-dependent material parameters. However, the observed reduction in PCE with high Cs concentration is consistent with experimental trends, despite these limitations. This reinforces our confidence that the efficiency loss is predominantly due to the increasing trap density, rather than modeling anomalies [80,83,98].

The perovskite absorber layer was tuned at 750 nm to achieve an equilibrium between light absorption and charge collection. This thickness considers the absorption depth (α^−1^ = 500 nm for MAPbI_3_) while adhering to the standard charge collecting length (~1 µm). Thinner absorber layers (<500 nm) would compromise photocurrent production, but larger layers (>1 µm) may result in heightened bulk recombination. This thickness also preserves the structural integrity of the mixed-cation perovskite (MA_0.5_Cs_0.5_PbI_3_) under environmental stress [79,85,92].

Similarly, the optimum doping level of 10^20^ cm^−3^ in the TiO_2_ layer was obtained from simulation to enhance device performance via many processes. The substantial doping markedly decreases the series resistance of the electron transport layer, directly enhancing our attained high fill factor (82.81%) and open-circuit voltage (0.92 V) by optimizing electron extraction efficiency. Secondly, doping elevates the Fermi level of TiO_2_ towards its conduction band, enhancing energy-level alignment at the TiO_2_/perovskite interface and reducing energy losses. This doping concentration effectively passivates defects in the TiO_2_ layer by mitigating oxygen vacancies, thereby diminishing trap-assisted recombination that may otherwise constrain device performance. The synergistic effects illustrate that meticulous tuning of doping density may markedly improve the overall photovoltaic efficacy of perovskite solar cells [38,39,42].

Simulated power conversion efficiency (PCE) of 18.53% for MA_0.5_Cs_0.5_PbI_3_ is consistent with actual findings for mixed MA-Cs systems (e.g., 17–19% PCE as reported by Ref. [87]), while recognizing the superior efficiencies (22.89%) attained in pure FAPbI_3_ devices via sophisticated interface engineering [98]. The primary difference is in the optimization priorities: Gao et al.’s coherent FAPbI_x_Cl_3−x_ buried interface specifically addresses interfacial defect passivation, enhancing V_oc_ from 1.01 V to 1.10 V, while our Cs^+^ doping strategy emphasizes bulk crystal stabilization, increasing V_oc_ from 0.85 V (MAPbI_3_) to 0.92 V via lattice hardening and grain boundary passivation. Both methodologies fundamentally focus on trap-state reduction as the primary means to enhance performance, whether via interfacial [98] or bulk (this simulation work) defect mitigation.

## 4. Conclusions

This work uses a series of simulations to investigate the optimization of mixed-cation MA_1−x_Cs_x_PbI_3_-based perovskite solar cells by evaluating the impact of cesium content and other parameters (such as thickness and doping) on the photovoltaic responses of the optimal devices. It is observed that Cs content, thickness, and doping density are critical factors in enhancing overall photovoltaic performance. The absorbing layer MA_1−x_Cs_x_PbI_3_ with x = 0.5, particularly the MA_0.5_Cs_0.5_PbI_3_ composition, exhibits an open-circuit voltage (V_oc_) of 0.92 volts, a fill factor of 82.81%, a short-circuit current density (J_sc_) of 24.22 mA/cm^2^, and a maximum efficiency of 18.53%. The results show that a balanced composition of Cs achieves the best performance; this may reduce defect density, strengthen crystal structure, and increase resistance to environmental elements such as temperature and humidity. With a TiO_2_ and Spiro-OMeTAD doping density of almost 10^20^ cm^−3^, the ideal thicknesses are 10–30 nm for the TiO_2_, 10–20 nm for the Spiro-OMeTAD, and 750 nm for the perovskite absorber layer, thus enabling enhanced charge extraction and reduced recombination losses. Because of a rise in trap states, elevated cesium concentration (x > 0.5) can reduce power conversion efficiency, so emphasizing the need for careful compositional adjustment. These results offer fundamental understanding for the design of strong and very efficient perovskite solar cells, so enabling their extensive commercialization and global shift to sustainable energy sources. Future studies must give experimental validation of these simulations top priority and look at flexible stabilization mechanisms to precisely match laboratory performance with useful application.

## Figures and Tables

**Table 1:** A list of the required simulation parameters utilized in this study is given below. The simulation parameters are extracted from reported literature, and their references are given in the table.

						MA_1−x_Cs_x_PbI_3_				
Photovoltaic Parameters	Unit	Symbol	ITO	TiO_2_	Spiro-MeOTAD	x = 0	x = 0.25	x = 0.5	x = 0.75	x = 1
Thickness	nm	Th	100	100	150	300.0	300.0	300.0	300.0	300.0
Energy Band Gap	eV	Eg	3.6	3.26	2.9	1.6	1.6	1.6	1.7	1.7
Electron Affinity	eV	Χ	4.1	4.2	2.2	4.00	3.98	3.95	3.93	3.90
Dielectric Permittivity		Ε	10	10	3.5	6.5	6.4	6.2	6.0	6.0
Effective Density of States at Conduction Band	cm^−3^	N_C_	2 × 10^18^	2.2 × 10^18^	2.2 × 10^18^	1 × 10^20^	1.5 × 10^20^	1.8 × 10^20^	2.1 × 10^20^	2.5 × 10^20^
Effective Density of States at Valence Band	cm^−3^	N_V_	1.8 × 10^19^	1.8 × 10^18^	1.8 × 10^19^	8 × 10^20^	7 × 10^20^	5 × 10^20^	4 × 10^20^	2.5 × 10^20^
Hole Thermal Velocity	cm/s	V_h_	1 × 10^7^	1 × 10^7^	1 × 10^7^	1 × 10^7^	1 × 10^7^	1 × 10^7^	1 × 10^7^	1 × 10^7^
Electron Thermal Velocity	cm/s	Ve	1 × 10^7^	1 × 10^7^	1 × 10^7^	1 × 10^7^	1 × 10^7^	1 × 10^7^	1 × 10^7^	1 × 10^7^
Electron Mobility	cm^−2^/V·s	µ_e_	50	20	1 × 10^−4^	50.0	44.0	38.0	32.0	25.0
Hole Mobility	cm^−2^/V·s	µ_h_	75	10	1 × 10^−4^	50.0	44.0	38.0	32.0	25.0
Uniform Shallow Donor Doping	cm^−3^	N_D_	1 × 10^19^	1 × 10^17^	0	0.0	0.0	0.0	0.0	0.0
Uniform Shallow Acceptor Doping	cm^−3^	N_A_	0	0	1 × 10^18^	1 × 10^13^	1 × 10^13^	1 × 10^14^	1 × 10^15^	1 × 10^15^
Defect Density	cm^−3^	N_T_	1 × 10^15^	1 × 10^15^	1 × 10^15^	1 × 10^15^	1 × 10^15^	1 × 10^15^	1 × 10^15^	1 × 10^15^
References			[44]	[44,45,46,47]	[38,47,48,49]	[38,49,50,51,52,53,54,55]				

**Table 2:** Defect parameters used in simulations for each device.

Defect Parameters	Unit	TiO_2_/MA_1−x_Cs_x_PbI_3_	MA_1−x_Cs_x_PbI_3_/Spiro OMeTAD	MA_1−x_Cs_x_PbI_3_
Defect Type	**-**	Neutral	Neutral	Neutral
Capture Cross-section for Electron and holes	cm^−2^	1 × 10^−14^	1 × 10^−14^	1 × 10^−14^
Energetic Distribution	-	Single	Single	Gaussian
Energy Level With respect to E_v_	eV	0.6	0.6	0.65
Characteristics Energy	eV	-	-	0.1
Total Density	cm^−3^	1 × 10^14^	1 × 10^14^	1 × 10^14^

**Table 3:** Comparison of published experimental photovoltaic results from mixed-cation perovskite solar cells.

Year	Absorber	HTL Material	ETL Material	V_oc_ (V)	J_sc_ mA·cm^−2^	FF (%)	PCE (%)	Ref.
2025	MA_0.5_Cs_0.5_PbI_3_	Spiro-OMeTAD	TiO_2_	0.92	24.22	82.8	18.53	This study
2025	FAPbI_x_Cl_3−x_ (with interface engineering)	-	SnO_2_	1.10	24.89	80	22.89	[80]
2023	FA_0.5_Cs_0.5_PbI_3_	HTL Free	PCBM	0.98	22.63	75	16.72	[81]
2023	FA_0.83_Cs_0.17_Pb(I_0.6_Br_0.4_)_3_	PTAA	C_60_	1.25	17.2	79	17.0	[82]
2023	CsPbI_2.25_Br_0.75_	PTAA/MoO3	SnO_2_/ZnO	1.35	16.49	74.0	16.4	[9]
2022	Cs_0.17_FA_0.83_PbI_1.8_Br_1.2_	Spiro-OMeTAD	SnO_2_	1.15	18.47	71	15.07	[83]
2021	FA_0.9_Cs_0.1_Pb(I_0.94_Br_0.06_)_3_	NiO	C_60_	1.01	22.7	64	14.8	[84]
2021	FA_0.9_Cs_0.1_Pb(I_0.94_Br_0.06_)_3_	NiO	C_60_	1.03	22.2	72	16.5	[85]
2021	FA_0.9_Cs_0.1_Pb(I_0.94_Br_0.06_)_3_	NiO	C_60_	1.03	21.0	62	13.5	[84]
2021	FA_0.9_Cs_0.1_Pb(I_0.94_Br_0.06_)_3_	NiO	C_60_	1.08	22.0	65	13.5	[84]
2020	MA_0.5_Cs_0.5_PbI_3_	HTL-free	TiO_2_	0.86	9.31	32	2.56	[86]
2014	Cs_0.1_MA_0.9_PbI_3_	Spiro-OMeTAD	PCBM	1.05	10.10	73	7.68	[85]
2016	MA_1−x_Cs_x_PbI_3_	Spiro-OMeTAD	TiO_2_	-	-	-	-	[87]
2017	MA_0.85_Cs_0.15_PbI_3_	Spiro-OMeTAD	TiO_2_	1.05	20.88	69	15%	[88]

**Table 4:** Summary of the effects of Cs content on the photovoltaic parameters of MA_1−x_Cs_x_PbI_3_ solar cells.

Cs Content (x)	Short-Circuit Current (mAcm^−2^)	Open-Circuit Voltage (Volts)	Full-Factor (%)	Power-Conversion Efficiency (%)	Comments
0 (Pure MA)	Moderate	Low	Moderate	Low	Instability & low Voc
0.5	High	Higher	High	Highest	Optimal balance
0.75	Moderate	Highest	High	Moderate	Jsc begins to drop
1.0 (Pure Cs)	Low	Lower than 0.75	Lower	Lowest	Likely phase instability

## Data Availability

Available on request.
